# Integrin-Linked Kinase (ILK) Regulates Urinary Stem Cells Differentiation into Smooth Muscle via NF-*κ*B Signal Pathway

**DOI:** 10.1155/2021/6633111

**Published:** 2021-03-27

**Authors:** Liang-liang Huang, Jun-hong Deng, Jing-xuan Xie, Zi-bin Lin, Hui Jiang, Bin Ouyang, Jian-ming Liu, Yan-ni Wei, Zhou-da Cai

**Affiliations:** ^1^Department of Andrology, Guangzhou First People's Hospital, School of Medicine, South China University of Technology, Guangzhou 510180, China; ^2^Department of Urology, Peking University Third Hospital, Andrology, Beijing 100191, China

## Abstract

**Objectives:**

Urinary stem cells (USCs) have the capacity for unlimited growth and are promising tools for the investigations of cell differentiation and urinary regeneration. However, the limited life span significantly restricts their usefulness. This study is aimed at exploring the effect of integrin-linked kinase (ILK) on the smooth muscle cells (SMCs) differentiation of the dog USCs and investigating its molecular mechanism.

**Methods:**

An immortalized USCs cell line with the molecular markers and biological functions was prepared. After successfully inducing the differentiation of USCs into SMCs, the expression level of the unique key factor and its mechanisms in this process was determined through real-time polymerase chain reaction, Western blot, or Immunofluorescence staining.

**Results:**

We found that high cell density promoted USCs differentiation SMCs, and ILK was necessary for USCs differentiation into SMCs. Knocking down ILK decreased the expression of SMCs specific-marker, while using a selective ILK agonist increased the expression of SMCs specific-marker. Furthermore, ILK regulated SMCs differentiation in part through the activation of NF-*κ*B pathway in USCs. A NF-*κ*B activity assay showed overexpression of ILK could significantly upregulate NF-*κ*B p50 expression, and NF-*κ*B p50 acts as downstream signal molecular of ILK.

**Conclusion:**

High cell density induces the differentiation of USCs into SMCs, and ILK is a key regulator of myogenesis. Furthermore, NF-*κ*B signaling pathway might play a crucial role in this process.

## 1. Introduction

Urinary stem cells (USCs) are pluripotent stem cells derived from voided urine [1, 2]. USCs can be abundantly and noninvasively obtained using a safe and low-cost procedure. It has been proved that USCs have the capacity for unlimited growth, self-renewal, and differentiation toward multiple cell lines including endothelial, osteogenic, adipogenic, and neural lineages [3]. Due to their high differentiation potential, USCs could be an excellent alternative cell source in cell-based therapies for many diseases. Literatures have demonstrated that USCs can differentiate into smooth muscle cells (SMCs) in response to growth factor simulation [4]. However, how exactly USCs differentiate into SMCs remains unknown, which further limits the possibility of clinical appliance. Importantly, understanding the molecular mechanisms of this differentiation assumes a new urgency.

Integrin-linked kinase (ILK), a serine-threonine protein kinase, is a crucial mediator which could transfer integrin signals from the extracellular matrix. Previous studies have proved that ILK plays essential roles in cell differentiation, adhesion, invasion, spreading, polarity, migration, proliferation, and survival [5, 6]. ILK has a vital function during fetal development and tissue homeostasis, and ILK dysregulation has been associated with cardiomyopathies and different types of cancer in humans [7–9]. In addition, it is widely accepted that ILK is well appreciated for its roles in stemness and metastasis [10, 11]. ILK was investigated for its potential as a nodal signal integrator for microenvironmental cues in survival pathway activation [12]. Previous study shows that mesenchymal stem cells induced by multiple myeloma cells differentiate into SMCs, which depends on the upregulation of ILK expression [13]. In our previous proteomics analysis, we observed that the protein levels of several proteins, including ILK, were significantly increased in the differentiating USCs; during early SMCs differentiation, we hypothesized that ILK could have a significant effect on USCs differentiation into SMCs. This suggests that ILK may influence USCs behavior in SMCs differentiation.

In the present study, we identified an immortalized dog USCs cell line, with the certain molecular markers and biological functions; our data displayed that USCs hold SMCs differentiation potential, particularly induced in cell-cell adhesion, and this process was mediated through ILK. More importantly, the results demonstrate that ILK stimulates USCs to differentiate into SMCs by activation of the NF-*κ*B signal pathway.

## 2. Materials and Methods

### 2.1. Isolation and Culture of Primary Dog USCs

Beagle Dogs (male, 3 months old) were obtained from Guangzhou General Pharmaceutical Research Institute and housed in pathogen-free conditions with constant temperature, humidity, and 12 h light/12 h dark cycle. All animal experiments were conducted in accordance with the guidelines of the Animal Experiment Committee of School of Medicine, South China University of Technology, Guangzhou, China. Methods that USCs were collected and expanded from urine were described previously with minor modifications [1]. Briefly, urine samples were collected from 6 Beagle Dogs, and each sample was centrifuged at 500 g for 5 minutes to collect the cells. The pellet was washed in buffer containing 1X Dulbecco's phosphate-buffered saline (DPBS) with Penicillin/Streptomycin, 0.5 mg/ml Amphotericin B. These cells were gently resuspended with primary culture medium composed of keratinocyte-serum free medium and progenitor cell medium in a 1 : 1 ratio. Cells were then plated to 24-well plates coated with 1% gelatin solution. During the first 72 hours, 1 ml primary culture medium was changed. Then, medium was changed and REGM was added until cells were expanded above 70% of the plate. USCs were plated at low density (4 × 10^3^ cells/cm^2^) and high density (40 × 10^3^ cells/cm^2^) in primary culture medium. After 48 hours, nonadherent cells were removed and fresh medium replaced the existing culture medium. After 10 days, the cells would be used in follow-up experiments.

### 2.2. Gene Expression and Quantitative RT-PCR (qRT-PCR)

qRT-PCR was performed to measure the gene expression from USCs cultured in different medium. Total RNA was isolated using the TransZol Up plus RNA Kit (ER501-01, TRANSGEN BIOTECH, China). The RNA was then reverse-transcribed to single-stranded cDNA using the PrimeScript RT Master Mix kit (Takara Bio). qRT-PCR analysis was performed using the ABI PRISM 7500 Sequence Detection System (Applied Biosystems, Foster City, California, USA) and the SYBR Green Real-time PCR Master Mix. All reactions were run in triplicate in three independent experiments. The primers used are listed.

### 2.3. Protein Extraction and Western Blot

Western blot was performed to quantify protein expression. Briefly, USCs were lysed and total proteins were separated by protein extraction containing proteasome inhibitor. The protein concentrations in the lysates were measured using the BCA Protein Assay Kit (Thermo Scientific, 23228, Rockford, IL). After incubation with primary antibodies and secondary antibodies in blocking buffer, targeted proteins were imaged with the ECL Plus Western Blot Detection System (GE Healthcare, UK). The immunoreactive bands were quantified by scanning densitometry software (NIH, Bethesda, MD, USA), and protein expression levels were normalized to those of a housekeeping gene, GAPDH. All the experiments were repeated three times over multiple days.

### 2.4. Flow Cytometry

USCs were detached with trypsin (Gibco-BRL) and disrupted as single-cell suspensions at an appropriate concentration. Tubes were labeled with monoclonal antibodies against mouse CD24, CD29, CD31, CD34, CD45, CD90, CD105, CD133, and SSEA-4 according to the instructions of the Human MSC Analysis Kit (BD Biosciences) on ice in the dark for 30 minutes. After washing with stain buffer and resuspended in 500 mL PBS, samples were analyzed using FACSCalibur flow cytometer (Becton Dickinson).

### 2.5. ILK-siRNA Transfection

We used validated small interfering RNA (siRNA) (#AM16708, Invitrogen; Thermo Fisher Scientific, Inc) directed against ILK messenger RNAs (mRNAs) according to the manufacturer's instructions. Briefly, USCs were seeded on 6-well plates and cultured to 50% confluence; these cells then were transfected with 100 nM of siRNA for ILK or siRNA-negative control (#AM4611, Invitrogen; Thermo Fisher Scientific, Inc). Cells were harvested and analyzed 48 hours after transfection in medium lacking serum and antibiotics. For analysis of knockdown efficiencies, qRT-PCR or Western blotting was performed to observe the inhibition of ILK expression within 24 hours, and this knockdown was maintained for at least 72 hours following removal of the medium containing the siRNA.

### 2.6. Drug Treatment

To induce ILK overexpression, USCs were incubated with 0.4 *μ*mol/L LPTP (L-*α*-Phosphatidyl-D-myo-inositol3, 4, 5-triphosphate, dioctanoyl, sigma), which was a potent and selective ILK agonist. At 2 hours after drug treatment, cells and supernatant were collected for Western blot analysis and following experiments. Similarly, lipopolysaccharide (LPS) (St. Louis, MO, USA), a component of Gram-negative bacteria, was used to activate the NF-*κ*B pathway. The concentration of LPS was 100 ng/ml; after 24 hours of incubation, cells were collected for the following experiment.

### 2.7. Immunofluorescence Staining

USCs were fixed in 4% paraformaldehyde for 20 minutes at room temperature; after blocking for 1 hour at 37°C in blocking buffer (5% goat serum, 0.01% (*v*/*v*) Triton X-100 in PBS), samples were incubated overnight at 4°C with the primary antibodies: ILK (ab76468, Abcam, 1 : 200), Desmin (ab32362, Abcam, 1 : 100), and SMN (ab5831, Abcam, 1 : 100). After washing with PBS, samples were conjugated with secondary antibody (ab150077, Abcam, 1 : 500; or ab150113, Abcam, 1 : 500), followed by staining with DAPI (25 mg/ml in TNE buffer: 10 mmol/l Tris-HCl, 2 mol/l NaCl, 1 mmol/l EDTA, pH 7.4; Molecular Probes). Images were captured with a FV1000 laser scanning confocal microscope (Olympus, Japan) according to the manufacturer's instructions.

### 2.8. Statistical Analysis

All values are expressed as mean ± standard deviation (SD). Statistical differences were evaluated using one-way ANOVA and the Student's *t*-test where applicable. Statistical analysis was performed using SPSS Statistics 20. *P* values smaller than 0.05 were accepted as significant.

## 3. Results

### 3.1. USCs Isolated from Urine of Dogs, and High Cell Density Promoted USCs Differentiation into SMCs

**Figure 1:**
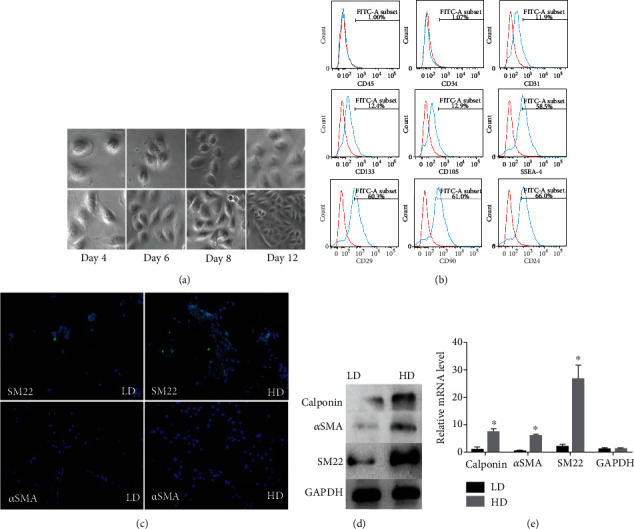
(a–e) USCs isolated from urine of dogs and high cell density promoted USCs differentiation into SMCs. Representative images of dog USCs expansion on days 4, 6, 8, and 12 at low density (LD) or high density (HD) (a). Flow cytometric analysis reveals CD surface antigen expression on dog USCs; pluripotent stem cell markers (SSEA-4, CD29, CD90, CD24) were strongly positive; CD31, CD133, and CD105 were weakly positive; hematopoietic cell marker CD45 and endothelial lineage markers CD31 and CD34 were negative (b). USCs were seeded at low density (LD) or high density (HD), immunostaining for SM22 and aSMA. Nuclei were counterstained with DAPI (blue) (c). Western blot and qRT-PCR for calponin, aSMA, and SM22 were quantified to show relative protein and mRNA levels in LD and HD USCs, respectively (c, d).

Progenitor cells isolated from the urine of dogs and were cultured in high and low density, respectively (Figure 1(a)). Flow cytometry was used to detect marker expression of these cells; we found that USCs were strongly positive for pluripotent stem cell markers SSEA-4, CD29, CD90, and CD24 and weakly positive for CD31, CD133, and CD105. In addition, the general hematopoietic cell marker CD45 and endothelial lineage markers CD31 and CD34 were negative (Figure 1(b)). To test whether cell-cell adhesion could affect the differentiation of USCs towards the SMCs, USCs were plated at low density and high density. The result of immunostaining showed that the myogenic protein levels of *α*-smooth muscle actin (*α*SMA) and SM22 were increased obviously (Figure 1(c)). Furthermore, the protein and mRNA levels of the calponin, *α*SMA, and SM22 were detected by Western blot and qRT-PCR; we found that the expression of these myogenic markers was obviously increased in high-density cultures (Figure 1(d) and Figure 1(e)). These results demonstrate that high cell density could promote USCs differentiation into SMCs.

### 3.2. High Cell Density Culture Increased ILK Expression

**Figure 2:**
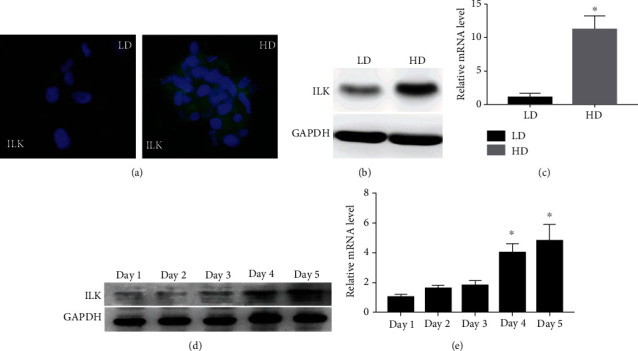
(a–e) High cell density culture increased ILK expression. Immunostaining for ILK in high density (HD) and low density (LD) cultures (a). Western blot and qRT-PCR for ILK were quantified to show relative protein and mRNA levels in LD and HD USCs, respectively (b, c). Expression of ILK proteins and mRNA levels over a period of 5 days was evaluated by Western blot and qRT-PCR (d, e).

In our previous proteomics analysis, we observed that the protein levels of several proteins, including ILK, were significantly increased in the differentiating USCs during early SMCs differentiation. To further confirm these finds, we conducted the following experiment. After 10 days of culture, immunostaining showed that expression of ILK was significantly higher in high cell density than low cell density (Figure 2(a)). Western blot showed that the protein level of ILK increased in cells cultured at high density compared with those cultured at low density (Figure 2(b)). In agreement, qRT-PCR showed that ILK mRNA level also increased (Figure 2(c)). Finally, the level of both proteins and mRNA of ILK was found to be upregulated during SMCs differentiation from USCs as cell density increased over time in culture, reaching a maximum by day 5 (Figures 2(d) and 2(e)).

### 3.3. ILK Promotes the Differentiation of USCs to SMCs

**Figure 3:**
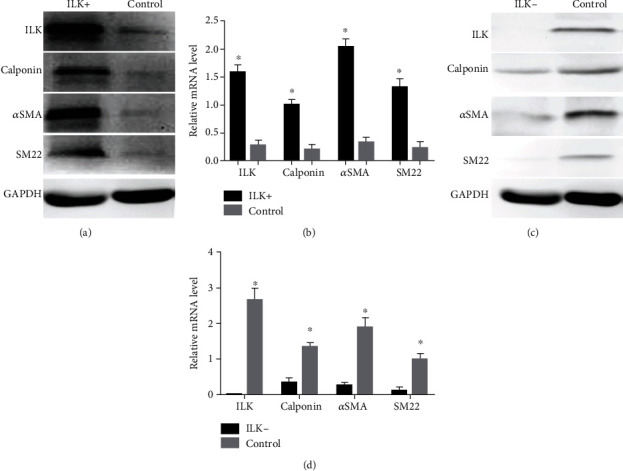
(a–d) ILK promotes the differentiation of USCs to SMCs. Western blot and qRT-PCR of ILK, calponin, aSMA, SM22 in USCs with LPTP (ILK+), and control USCs were quantified to show relative protein and mRNA levels (a, b). Similarly, Western blot and qRT-PCR of ILK, calponin, aSMA, SM22 in USCs with knocking down ILK (ILK-), and control USCs were quantified to show relative protein and mRNA levels (c, d).

To study the effect of ILK on the myogenic differentiation of USCs, these cells were incubated in differentiation media with or without 0.4 *μ*mol/L LPTP, which was a potent and selective ILK agonist. The results showed that LPTP could increase the expression of ILK; Western blot and qRT-PCR showed that calponin, *α*SMA, and SM22 protein and mRNA levels were upregulated after the incubation of LPTP, respectively (Figures 3(a) and 3(b)). Additionally, siRNA was employed to knockdown ILK. As shown by Western blotting and qRT-PCR, knocking down ILK had profound effects on USCs differentiation, calponin, *α*SMA, and SM22 protein and mRNA levels were diminished significantly (Figures 3(c) and 3(d)). Taken together, these data indicate that ILK is necessary for USCs to differentiate into SMCs.

### 3.4. The NF-*κ*B Signaling Pathway Involved in the ILK Stimulated Differentiation of USCs to SMCs

**Figure 4:**
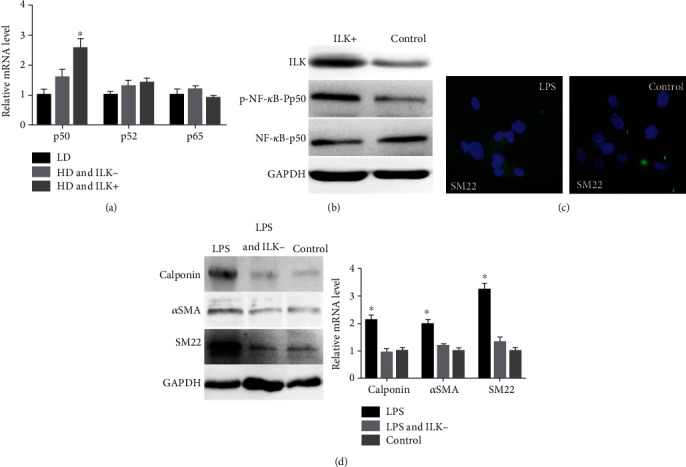
(a–d) The NF-*κ*B signaling pathway involved in the ILK stimulated differentiation of USCs to SMCs. qRT-PCR detected the mRNA expression levels of NF-*κ*B p50, NF-*κ*B p52, and NF-*κ*B p65 in low density (LD) and high density (HD) (a). Protein levels of ILK, p-NF-*κ*B p65, and NF-*κ*B p65 in USCs with LPTP (ILK+) and control USCs were detected using Western blot analysis (b). Lipopolysaccharide (LPS), a component of Gram-negative bacteria, which is used to activate the NF-*κ*B pathway of USCs, immunostaining for SM22 in USCs with LPS and control USCs (c). The expression of SMC-specific markers (calponin, aSMA, SM22) in USCs with the effects of NF-*κ*B activator LPS, LPS, and knocking down ILK (ILK-), or nothing, were detected by Western blot and qRT-PCR (d).

As a previous study indicated that SMCs differentiation involves the NF-*κ*B signaling pathway [14]; therefore, we wondered if ILK could promote SMCs differentiation gene expression through regulations of this signaling pathway. By examining the mRNA expression levels of NF-*κ*B, we identified that overexpression of ILK can significantly upregulated NF-*κ*B p50 expression, while no such effects on NF-*κ*B p52 and p65 expression (Figure 4(a)). After the incubation of LPTP, we found that overexpression of ILK significantly unregulated the phosphorylation level of NF-*κ*B component p50, without affecting the total p50 protein level (Figure 4(b)). In addition, we observed LPS, which could activate the NF-*κ*B pathway, can almost increase ILK-induced smooth muscle-specific markers expression. Immunostaining showed that LPS treatment significantly upregulate the level of SM22 (Figure 4(c)). Moreover, LPS could increase the expression of the smooth muscle-specific markers protein and mRNA levels. However, the effect of LPS on smooth muscle-specific markers does not occur if ILK is knocked down (Figure 4(d)). These results implied that the NF-*κ*B signaling pathway involved in the ILK stimulated differentiation of USCs to SMCs.

## 4. Discussion

Stem cell-based therapy and tissue engineering have been widely used in regenerative medicine. Among stem cells, USCs have relatively superior ethical issues and availability for clinical utility because they can be obtained through minimally invasive procedures [15]. USCs have already been applied to direct SMCs differentiation [16]. However, the underlying molecular mechanism and efficiency of SMCs differentiation still need more study. ILK has been proved to be an important role in SMCs development [17–19]. Here, the SMC differentiation capacity of ILK in USCs was tested.

Our findings show that USCs are considered as an alternative cell source capable of SMCs differentiation. More importantly, our work reveals a novel role for ILK in USCs differentiation into SMCs. Knocking down ILK decreased the expression of SMCs proteins and mRNA levels, or using a potent and selective ILK agonist increased the expression of SMCs protein and mRNA levels, suggesting ILK is essential for enhancing their SMCs differentiation efficiency.

The precise regulation of USCs differentiation is always difficult, the current research direction is to add some exogenous differentiation factors, but the effect is not satisfied. Previous studies have implicated that mechanical forces through the surface proteins regulate stem cell differentiation [20–22], our results agree on these observations. We found that high cell density culture can induce the differentiation of USCs into SMCs, but not low cell density. This situation may have two possible reasons. Firstly, it is due to changes in the cell's microenvironment, high cell density cultures of USCs may result in the increased production of lactic acid and the absence of oxygen, this can further change the microenvironment of USCs, and it could induce DNA damage and genome instability. Eventually, these microenvironment changes may lead to complex USCs differentiation changes. Secondly, high cell density cultures can induce cell-cell adhesion, which promotes cell-cell interactions and paracrine effects of surrounding cells; this situation may ultimately regulate USCs differentiation. Our result means that it will be a new direction that high cell density cultures are helpful for USCs differentiation; we can regulate the differentiation by modulating the density of USCs, even in the absence of differentiation factors. It will be a potential implication for the development of strategies to understand and control USCs fate decisions.

As a critical adaptor and mediator protein in the normal development of numerous tissues [17], ILK also plays important role in regulating cell adhesion junction, and ILK is critical for the invasiveness of human ovarian cancer cells [23]. Interestingly, we found that ILK could promote the differentiation of USCs to SMCs in the present study. ILK is widely distributed in a wide variety of mammalian cell types and tissues; its most important function may be to serve as an adaptor protein modulating cell-matrix interactions [24]. Previous study showed that ILK plays an important role in the hypoxia-induced phenotypic transition of pulmonary artery smooth muscle cells [25]. Furthermore, ILK plays a pivotal role in regulating mesenchymal stem cell survival and VEGF expression [26]. ILK is also found as a central component to ensure linkage between integrins and the actin cytoskeleton [27]. However, previous studies mostly focused on the important role for ILK in therapeutic strategy to malignancy; our work reveals an unknown role for ILK in the process of USC differentiation into SMCs. This is the first study implicating ILK in USC differentiation into SMCs.

We further examined the mechanism of ILK in SMCs differentiation of USCs. Our findings show that ILK regulated SMCs differentiation in part through the activation of NF-*κ*B pathway in USCs. Overexpressions of ILK can significantly upregulated NF-*κ*B p50 expression, while no such effects on NF-*κ*B p52 and p65 expression. Previous study has shown that ILK can regulate various oncogenic signaling pathways involved in modulation of the immune system with activator of NF-*κ*B signaling pathways, and inhibition of ILK could disturb the NF-*κ*B signaling loop [28, 29]. In addition, ILK could regulate cell metastasis through NF-*κ*B signaling in lung cancer [30]. These findings reveal that ILK acts as an intermediary effector of the NF-*κ*B feedback loop. In our study, we activated NF-*κ*B pathway by LPS in USCs, ILK-induced SMCs specific markers expression was significantly upregulated. These results support such notion that ILK could regulate SMCs differentiation gene expression, partially through modulating of one or more transcriptional factors such as NF-*κ*B p50.

Interestingly, we found that LPS alone could not increase the smooth muscle-specific markers expression without ILK activation, implying that the function of NF-*κ*B signaling pathway in SMC differentiation depends on ILK activation. This observation demonstrates that there is a complex interplay between NF-*κ*B and ILK in USCs differentiation, and it is highly likely that there are additional ILK-dependent signaling pathways, which provided a new perspective to understand the differentiation of USCs. To verify this hypothesis, further studies are underway in our laboratory.

## 5. Conclusion

Collectively, these observations support the general concept that high cell density culture induces the differentiation of USCs into SMCs, and ILK is a key regulator of myogenesis. Furthermore, we found that the NF-*κ*B signaling pathway might play a crucial role in this process. This research may hopefully direct the biomaterial modification and biofunctionalization.

## Data Availability

Data will be available on reasonable request.
